# Paramedics’ perceptions of the care they provide to people who self-harm: A qualitative study using evolved grounded theory methodology

**DOI:** 10.1371/journal.pone.0205813

**Published:** 2018-10-17

**Authors:** Nigel Rees, Alison Porter, Frances Rapport, Sarah Hughes, Ann John

**Affiliations:** 1 Pre Hospital Emergency Research Unit (PERU), Institute of Life Sciences, Swansea University, Swansea, Wales, United Kingdom; 2 Institute of Life Sciences, Swansea University Medical School, Swansea, Wales, United Kingdom; 3 Australian Institute of Health Innovation, Macquarie University, Sydney, New South Wales, Australia; 4 Abertawe Bro Morgannwg University Health Board, Princess of Wales Hospital, Bridgend, Wales, United Kingdom; 5 Farr Institute, Swansea University Medical School, Swansea, Wales, United Kingdom; University of Toronto, CANADA

## Abstract

**Background:**

Self-harm (SH) accounts for over 5% of the workload of emergency ambulance services, and therefore Paramedics are often the first health professional in contact with people who SH. The authors of this paper have reported elsewhere the significant gaps in our understandings which exist surrounding this early care interaction, and some of the challenges paramedics and opportunities in paramedic care for people who SH. This study aimed to explore paramedics’ perceptions of caring for those who SH using Evolved Grounded Theory Methodology.

**Methods:**

This study took place between 2014–2016 in one UK ambulance service covering a population of three million people. Semi structured interviews were conducted, purposively sampling paramedics until saturation was reached. Interviews were recorded, transcribed verbatim, and coded through open, axial, and selective levels of coding, identifying the Basic Social Process (BSP) and developing a Grounded Theory. A second researcher (SH) independently reviewed early results, which were also member-checked with participants.

**Results:**

Eleven paramedics were interviewed. The following six categories emerged: *Context; Judgements and values; Isolation and system failure; Managing risk; Competence at the boundary of mental and physical health needs; Professional*, *legal and ethical tensions*. The BSP *Decision making in a context of risk* was identified. The final Grounded Theory that emerged was one of ‘*Wicked Complexity of paramedic care for people who SH*, which includes *usual factors* such as tiredness and frequent callers, *heightened* factors including lack of support and pathways, and *factors specific to SH* such assessing mental health and suicide risk.

**Conclusions:**

This study builds on a very small body of literature to have explored paramedic care for people who SH and has found that this care interaction provides uniquely complex challenges. The multiple influences within the categories defined in this study need considering conjointly when making improvements to care.

## Introduction

Self-harm (SH) is defined as an intentional act of self-poisoning or self-injury, regardless of the motivation or degree of suicidal intent [1]. For emergency ambulance services, SH patients make up 5.7% of all 999 calls [2], with up to half of patients making at least one repeat call [3]. In England, hospital admissions due to SH have risen over the last decade, from 91,341 in 2005, to 112,096 in 2015, with 98.8% of these being emergency admissions [4]. These figures do not reflect the true scale of SH, as only 10–20% of those who engage in the behaviour present to hospital, suggesting there is a hidden population of distressed individuals not accessing care [5]. The true scale of SH is estimated at 1 in 130 people, many of whom may have serious mental health problems but are reluctant to engage with services [6]. Appropriate management of people who SH is vital, since a history of SH is strongly associated with suicide [3,7].

Paramedics are often the first health professionals contacted following SH [8,9], and whilst paramedic guidelines [10] cover aspects of SH care, education for paramedics on SH management is limited. Calls have been made for qualitative research focusing on occupational groups, such as paramedics, to better understand care delivered to those who SH [1,7]. Despite this, reviews of the literature, Rees et al (2014 2015) [8, 11] have highlighted the limited nature of published evidence about care provided by paramedics for those who SH. This paper presents a qualitative study of paramedics’ perceptions of caring for people who SH to inform education, policy and practice.

### Aims

The aims of the study were to: 1. reveal and explore paramedics’ perceptions of caring for those who SH, 2. develop a theory on paramedics’ perceptions of caring for SH.

## Method

### Methodological approach

This study uses Evolved Grounded Theory Methodology (EGTM) [12,13], a derivative of Grounded Theory Methodology (GTM) [14]. EGTM takes a constructivist view, acknowledging multiplicity of perspectives, truths and realities through flexible, naturalistic data collection methods linked to analysis that considers the adaptability of human situations [12,13].

In line with EGTM, the study set out to generate theory. Based on the study aims, data were analysed using an inductive–deductive interplay approach. Induction is viewed as the key process in EGTM, with the researcher moving from the data to empirical generalisations, and on to theory-development. Strauss and Corbin (1998) [12] advocate such induction-deduction, via ongoing data comparison, where emergence is ensured by deduction strategies, followed by verification and elaboration from further data comparison. The researcher shapes data by interpretation, moving analysis beyond description. In order to monitor researcher preconceptions and undue influence on the analysis process and on theory development, the lead author (NR) was reflexive about having been a paramedic for twenty-eight years, and having cared for many people who had engaged in acts of SH, including witnessing deaths following acts of SH.

### Ethics

National Health Service (NHS) ethical approval was deemed unnecessary during proportionate review, as interviews were with existing staff and no changes to their practice were planned, nor was any contact made with patients. NHS Research and Development Permissions were sought and granted. The Economic and Social Research Council’s [15] framework for ethical standards was followed. Participants provided written informed consent.

### Setting, sampling and recruitment

The study was conducted between 2014 and 2016 in one UK ambulance service, covering a population of three million people. A poster requesting volunteer paramedics for the study was distributed to the whole ambulance service area. Following responses to the poster call, potential participants were selected using theoretical sampling methods, based on the characteristics of age, gender, years of paramedic experience, rural or urban setting, and educational development. These characteristics were identified by a systematic review and metasynthesis of the literature previously conducted by the researchers [8, 11] which revealed that attitudes to SH amongst health professionals are more positive in those prepared through higher education rather than vocational training [16,17], and also that older and more experienced health staff had more supportive attitudes, empathy and positive attitudes than younger and less experienced staff [18]. Geographical setting also had potential to influence paramedic perceptions of SH, as urban SH rates have been shown to be substantially higher than rural rates [19], whereas completed suicide rates are higher in rural than non-rural areas [20]. Ambulance work was historically male dominated, and the one published study on SH involving ambulance staff [21] found significantly more males expressed unfavourable attitudes towards SH than their female counterparts, however, one third of paramedics currently registered in the UK are now female [22]. The sample was therefore selected to represent these a balance of these issues. Sampled participants were provided with a pack including a consent form and an information sheet about the research.

An interview guide was developed, informed by the findings of a metasynthesis [11] and systematic review [8]. The interview guide consisted of semi-structured questions in line with the study’s aims and objectives, and explored: paramedics’ thoughts about the care they provided for people who SH; their perceptions of high or low standards of care; difficulties or barriers to providing appropriate care; and whether they were sufficiently informed and supported to deliver a high standard of care for people who SH.

Two pilot interviews were undertaken, one with a paramedic working in an urban area and the other with a paramedic working in a rural area. Only minor revisions were made to the interview guide following piloting, and pilot data were included in the main analysis. Subsequent participants were sampled based on emerging concepts in the data, to include participants with attributes more likely to reveal and explore insights through emerging concepts.

### Methods: Coding and analysis

NR conducted one-to-one interviews. These were audio-recorded and transcribed verbatim. Researcher’s notes, which added data about context and emotions expressed in interviews were taken. Data were collected until no new information was forthcoming, and thematic categories were saturated [12]. This included a second interview with two of the participants, to further explore insights revealed from all first-round interviews.

Transcriptions of interview recordings were read line by line, and coded using NVIVO (2015) V10 software [23]. The first stage was open coding, in which data were compared for similarities, differences and questions regarding emergent phenomena. Next, axial coding developed categories, taking concepts and thinking of how they could be subsumed under a higher-level heading. Finally, selective coding was an “*explication of the story line*” (Strauss & Corbin 1998, p. 148) [12] which involved identifying the basic social process (BSP) at work, around which all other categories revolved. Finally, theoretical constructions conceptualised the relationship among the three levels of coding, by weaving the fractured data back together again to form a Grounded Theory.

Transparency and trustworthiness in coding was observed by a second researcher (SH), independently reviewing the coding framework and then through discussion with the study team. Reflective notes were made by both researchers, Notes considered theory development and monitored the researchers’ influences on this process. In addition, the primary researcher returned to study participants to check their response to the researchers’ interpretation of data, using ‘member-checking’ techniques [24]; seven respondents agreed to take part in this process.

## Results

Out of twenty-six volunteers, eleven paramedics were interviewed. Participants’ experience ranged between one and thirty-eight years of frontline paramedic care. The cohort comprised three women and eight men; six had completed degree-level paramedic development courses, and five had undertaken traditional vocational training and five had received education specific to SH. The main researcher was known to all the participants, as he had been a prominent individual in the development of paramedic practice and education for many years in small group settings and large lectures. The paramedic community is a small professional group, and due to the main researchers’ length of service and support of education, he had on occasions crewed an ambulance with three of the participants, and as there is only one provider of higher education for paramedic in the study area, three of those developed through higher education had been taught by him. Table 1 presents this sample of study paramedics.

**Table 1:** Sample of study paramedics.

Gender	Male 8	Female 3	
Years EMS experience	1–3 years 3	3–10 4	>10 4
Age range	Up to 18 n = 0 19–26 n = 3	26-45n = 7 46–55 n = 0	56–60 n = 1 over 60 n0
Paramedic educational development	Higher Education n = 6	Traditional Paramedic Training n = 5	Specific SH training/Education n = 5

Through the axial coding process, six categories emerged to describe paramedics’ perceptions of caring for people who SH.

### Categories

#### Category 1: Context

Paramedics described the importance of the context of the encounter with people who SH at two phases in the process of care: pre-arrival, and on-scene care. The pre-arrival phase included contextual factors relating to the patient, such as the role of drugs or alcohol; those relating to the crew, such as how busy they were; and factors relating to the incident, such as who made the call.

Paramedics reflected on how pre-arrival contextual factors could sometimes prime them with negativity towards a person they were responding to following SH. Preconceptions (such as the stigma associated with SH) being amplified when responding to SH within the context of busy periods, when they themselves were tired:

*“Sometimes we are so browbeaten ourselves*, *or caught up in our own problems*, *overworked*, *underfed*, *whatever reason*. *These people are treated as a bloody nuisance*.*”* (Paramedic 4)

Paramedics also reported their frustration at their inability to respond to more serious calls whilst caring for those who SH:

*“Because they are taking us away from something more urgent*.*”* (Paramedic 3)

When ‘on-scene’, contextual factors continued to influence care, although crews now emphasised the importance of patient and incident related influences. These included the severity of injury; presence of others such as the family, police colleagues or bystanders; weapons; and alcohol impairing a patient’s capacity and decision-making:

*“Alcohol can affect somebody’s judgement*, *their mood*, *and you know*, *the way that they see things*, *and then they can be quite easily sort of wound up*.*”* (Paramedic 9)

Caring for patients who SH was described as often being a melancholic encounter tinged with sadness, shame and embarrassment. Paramedics reported that the presence of relatives could be problematic because of the “*embarrassment of the situation that [patients] find themselves in*.*”* (Paramedic 8). Paramedics learnt to manage the situation in response:

*“put[ting] the relative in the front*, *and that's generally when we find that your patient will talk to you more about the problems they find*. *They just don’t want friends/family knowing what they're going through*.*”* (Paramedic 4)

Shame and embarrassment in the presence of relatives may result in paramedics being prevented from gathering all the information they need to make an assessment and provide care appropriately; it may also make it less likely for patients to be left at home with relatives, especially in the absence of a full understanding of the situation prior to the SH.

#### Category 2: Judgements and values

Paramedics in our study revealed much about the personal aspects of the relationship between paramedics and patients who SH, including some attitudes which were negative and stigmatising. Paramedics admitted to internal tensions they experienced with people who SH:*“I don’t understand*, *inflicting pain on yourself”* (Paramedic 2). Paramedics recognised how important it was to gain understanding of why people SH:

*“The ‘whys’ the ‘hows’*. *Having a comprehension of what goes on in that person’s head to make them want to cut themselves*, *to make them want to put a rope around their neck*. *To make them want to take every tablet they can get their hands on*. *I think we need to understand the why behind that*.*”* (Paramedic 11)

Paramedics described frustration at the frequency of encountering SH patients—which could be “*two*, *three a night working in the city centre*” (Paramedic 2). They also felt frustration at responding to the same patient time and time again following SH, dealing with patients who “*become what we call regular”* (Paramedic 3); one paramedic used the pejorative term “*self harm regular repeat offenders”* (Paramedic 6). For some paramedics, the frequency with which they attended the same caller reduced their sympathy:

*“If you saw the same patient over and over and over again*, *and it's the same thing*, *you know*. *You can sort of*, *you know*, *get less sympathetic towards it like*, *you know*, *you can be more sort of hardened*…*Especially if you’re having an off day yourself*…” (Paramedic 9)

However, paramedics also reported positive aspects to having developed personal relationships with ‘regular’ SH patients, since these relationships helped to build trust and understanding, and their descriptions provided empathetic accounts which acknowledged the patient’s situation, and conveyed a sense of care:

*“Opens his neck up and that is bloody scary when he does that*, *there’s blood everywhere like and he’s bloody hysterical…he has learned not to be awkward with us mainly because we are not awkward with him… We know him quite well… I mean he’s just a person with issues”* (Paramedic 7)

#### Category 3: Isolation and system failure

Paramedics felt that increasingly they are the first point of contact for people who SH. They perceived there to be failures elsewhere in the health system which led to the ambulance service being contacted for mental health related issues, and suggested that serious mental health crises may have evolved from lack of access to earlier support. Paramedic 2 recalled how a person he attended with multiple mental health problems had tried accessing mental health pathways prior to an emergency call for a suicide attempt:

*“he had gone to his GP*, *he’d tried all that sort of stuff*. *He was desperate for help*. *Unfortunately he didn’t feel that the system was quick enough for him*. *So he tried to take his own life by hanging*.*”* (Paramedic 2)

Referral options available to paramedics were seen as limited, and not offering the kind of care which was most appropriate for the patient. In most cases their only option was to transport the person to the ED, and whilst they acknowledged some individuals may have injuries or medical problems which required ED treatment, they did recognise that for many, the ED may not be the best place. Powerful accounts were provided of the uncomfortable position paramedics found themselves in by not being able to offer what they felt was the right care:

*“The Emergency Department is often busy*, *noisy*, *can be confrontational*. *Staff don’t have time to deal with a patient on an individual basis*, *which is what's required*.*”* (Paramedic 6)

When faced with dealing with people who SH paramedics, reported feeling ill-equipped and unsupported, and in many cases *“lonely*.*”* (Paramedic 2). Paramedics described being unable to access appropriate care, including Crisis Resolution Teams:

*“Phoned* *[*ambulance*]* *control*, *phoned GP out of hours who said she needs to go in but can’t force her*, *Police tried ward*, *who wouldn’t take her cause she’d been drinking*, *called crisis team*…*forget it*.*”* (Paramedic 6)

#### Category 4: Managing Risk

Paramedics expressed concerns about the need to manage risks to patients and to themselves. They were particularly concerned about the risk of a patient going on to die by suicide, and these perceived risks influenced paramedics’ decision-making to ensure that patients who SH attend hospital, by force if necessary. Paramedics were anxious to be seen to be doing the right thing, not just for the patients’ sake but so as not to put their own jobs at risk:

*“A lot of paramedics … are extremely fearful of the way that they can be perceived…they're fearful of complaints and litigation against them*, *and fearful of losing their professional status*.*”* (Paramedic 2)

At its most extreme, this was expressed in a sense of ‘not on my watch’:

**“***I mean if he wants to kill himself then I can’t stop him doing that but I don’t particularly want him to kill himself when I’m involved*.” (Paramedic 1)

Paramedics also described concern about the risk of aggression or violence from patients who SH. The scene where somebody has self-harmed could be a threatening and dangerous environment, sometimes involving intoxicated patients, and implements used to SH which could be used as weapons against the paramedics or others. When faced with aggression, violence and potential assault with weapons, paramedics reported feeling concerned and vulnerable:

*“when you walk through the door and she is still holding the kitchen knife*, *and she says*, *you know*, *“I don’t want to live*, *you're not going to save me*,*” and she waves the knife at you then*, *obviously*, *there's a little bit of feeling of self preservation*, *and turning around and walking out the door”* (Paramedic 3)

In such situations paramedics valued the assistance from the police. Sometimes police were used in the detainment of a person who had self-harmed, but in most cases they were there to protect the safety of the ambulance staff, the patient and others. If they were not already deployed to the scene, they would often be called out by the paramedics:

*“I looked down and the patient had rolled over and she had a knife in her hand*, *but in like a dagger type action…*. *I was basically trapped… she was between the door and me*. *So we backed off*, *erm*, *we had to ring the police from the house phone to back us up… the patient was fine*, *we ended up taking her into hospital*.” (Paramedic 8)

Paramedics also described risk of further harm to the patients, including examples of further SH taking place during patient contact, which was upsetting and traumatic:

“*she smashed a bottle and stuck it into her groin*, *and was cutting her groin with the bottle*, *and it was quite*, *quite*, *quite nasty*. *She really bled out*, *so it was quite difficult to deal with*.” (Paramedic 9)

#### Category 5: Competence at the boundary of mental and physical health needs

Paramedics are used to assessing, prioritising, treating and referring patients across a range of presentations, making immediate decisions about care and treatment options. However, they reported a lack of confidence and competence in such decision making in relation to SH, which was seen as straddling the boundary of mental and physical health needs in a way which could be confusing. Paramedics revealed challenges over appropriate assessment, risk stratification, and referral for people who self-harm where “*there's no wound”* (Paramedic 2), compared with what were seen as more straightforward physical presentations:

*“there's no medical condition…you've got no tools…if you've got someone with a respiratory condition*, *you know*, *you've got tools in your bag that you can do something about it*.*”* (Paramedic 11)

Paramedics pointed out that their training and education focussed on technical and physical conditions such as trauma and cardiac presentations at the expense of mental health related issues. They suggested that better training should focus on:

*“how do we treat that sort of mental health issue rather than the physical injuries ‘cause I feel that we’re quite appropriately trained to do that”* (paramedic 8)

#### Category 6: Professional, legal and ethical tensions

Complex tensions were revealed by paramedics in applying challenging ethical and legal principles in clinical practice. Irrespective of the severity of injury from SH, suicidal intent was frequently assumed:

*“Anybody who causes self-harm then*, *you know*, *we automatically assume*, *and perhaps wrongly on occasions*, *that the intent is always an attempt at suicide*.*”* (Paramedic 9)

This assumption resulted in paramedics taking on a role as the preserver of life, setting up a tension between acting in the best interest of the patient, and responding to the patient’s wishes. Paramedic 4 explained the imperative to convey patients to hospital:

*“… whether it's right or wrong*, *if I believe*, *even half believe*, *that a person had taken an amount of medication that would seriously damage their health*, *and they were refusing to go in… I'd rather get into trouble ethically for taking somebody against their will than somebody die*, *and I left them there to die*.*”* (Paramedic 4)

All study paramedics reported an understanding of the law in relation to SH, in particular the Mental Health Act 1983 [25] and the Mental Capacity Act 2005 [26], but many also reported lacking confidence in interpreting and applying the law and described some of the tensions they experience. The MCA 2005 [26] asserts the rights of patients to refuse care if they wish–as long as they are deemed to have the capacity to make an informed decision. Paramedics feel as if they are put in the position of having to assess whether or not a patient has capacity.

Police have the power under the MHA 1983 [25] to support paramedics by detaining a patient so they can then be conveyed to hospital–but only if the patient is in a public place. A strategy of coaxing patients out of the home so this could happen was revealed independently by five paramedic participants:

*“A detention under Section 135 and 136 [of the MHA] needs to take place in a public place*. *Therefore*, *often the easiest way to do it*, *to prevent somebody*, *or to treat somebody with self-harm was normally to try to coax them into a public place where a police officer could take action*.*”* (Paramedic 6)

### Basic Social Process (BSP): Decision-making in a context of risk

*Decision-making in a context of risk *was identified as the BSP in paramedics’ care for people who SH. Paramedics had to make on-the-spot decisions about what the patient’s needs were, what care to provide, what onward referrals to make, whether or not conveyance to hospital would be appropriate, and how to handle the situation if the patient’s decision about care differed from the paramedic’s. In some cases they had to decide whether the patient should be regarded as having the mental capacity to make their own decisions about what should happen. All of these decisions–complex in themselves–were carried out in a context of risk, both in terms of immediate risk of further SH or of harm to the paramedic or others, and also in terms of future risk of SH and to the paramedic’s own professional status.

Risk and decision making, alongside anxiety about fault finding and aggressive disciplinary action, are familiar and significant issues across paramedic work [27,28], and the challenges associated with decision making in relation to mental health have been noted [29]. Paramedics were most concerned of the risk of a patient they had cared for dying by suicide. Whilst most people who SH do not go on to die by suicide, SH elevates risk of suicide, as people who SH are also 100 times more likely than the general population to die by suicide [7], presenting a clear link between SH and suicide, and any discussion around paramedic care for those who SH must recognise such a link. There are many other risk factors for suicide following SH; they include: being male, unemployed, bereaved, past SH, social isolation, physical and mental illness, alcohol and other substance misuse [30]. Indications of higher suicidal intent includes: SH being well planned or carried out in isolation, final acts, such as writing a note, and more violent method of self-harm [30]. Pompili et al (2013) [31] found that people with insomnia who SH and attend ED more frequently use violent methods and this phenomenon should be taken into serious consideration by Paramedics.

Risk prediction tools have been developed to support decision making in SH and suicide and are recommended for paramedic use [10]. However, Quinlivan et al (2017) [32] found that such scales performed no better, and sometimes worse, than clinician or patient ratings of risk, and recognised their limited clinical utility, leading to a waste in valuable resources. In line with guidelines [1], Quinlivan et al (2017) [32] advised that risk scales should not be used to determine patient management or to predict suicide, and along with NICE (2004) [7] advise they should not be relied upon.

Paramedics reported concerns over not conveying the person to the ED, and fear of harm coming to the patient should they be left at home. Though it has been suggested that somewhere between 19.4% and 40% of attendances at EDs in the UK are avoidable [33,34], there is lack of rigorous evidence on the safety and appropriateness of decisions to avoid ED by ambulance staff [35]. Concerns of harm coming to a patient who did not attend hospital is not specific to SH, with other studies identifying paramedics’ need to protect themselves from challenges by ‘covering their backs’ [36,37].

#### Grounded Theory (GT): Wicked complexity in paramedics’ care for people who self-harm

**Fig 1:**
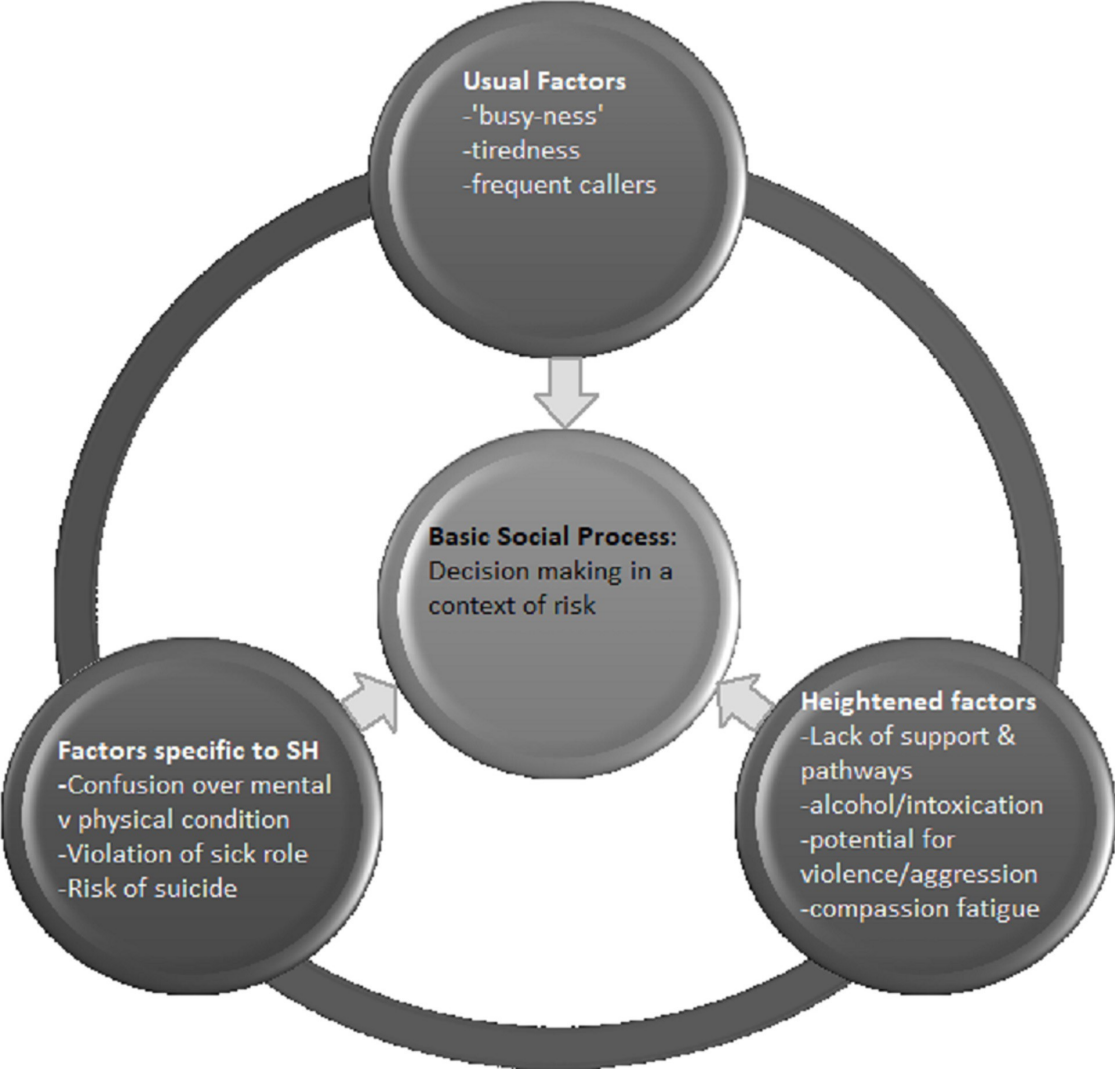
Overview of Grounded Theory (GT): Wicked complexity in paramedics’ care for people who self-harm.

The final stage in analysis was to derive a grounded theory (GT) describing paramedics’ care for people who self-harm, drawing on the BSP and the thematic categories presented above. The model in Fig 1 presents the GT: at its centre is the BSP of decision making in the context of risk, which is influenced by *usual factors*, such as tiredness and frequent callers, common to routine paramedic work; *heightened* factors including lack of support and pathways which are found to a greater degree in work with people who SH; and *factors specific to SH* such as assessing suicide risk.

We argue that paramedic care for people who SH is an example of ‘*wicked complexity’*. ‘Wicked’ issues [38] are those with built-in complexities, no clear definition of the problem, and a complex and sometimes changing set of external factors, as opposed to ‘tame’ problems which have a proper focus, appropriate definitions, and relevant information related to them. The factors presented below act individually and also in combination with each other. Each presents its own set of challenges. Fig 1 is an overview of the Grounded Theory (GT): Wicked complexity in paramedics’ care for people who self-harm.

#### Usual factors

Many of the issues raised by paramedics are commonly found in routine practice, regardless of the particular patient group, and these form a backdrop to decision making. Paramedics having a busy day might feel tired or stressed, and might also feel pressure to turn around jobs more quickly in order to be available for the next call. Due to the unplanned nature of emergency ambulance work, ‘busy-ness’ can vary day to day, but across the world demand for ambulance services has more than doubled in recent decades [39].

The frustration reported by paramedics in our study at attending the same person on a regular basis is a common problem in ambulance services, and although there is a high incidence of SH and mental health problems among frequent callers [40,41] the phenomenon of frequent calling is not confined to SH patients [4]. In recognition of the cost of responding to frequent callers, and the higher than average risk of conveyance, hospitalisation and death in this group [42], attention is being focused across the UK and internationally on finding more effective ways to manage this group [40,43,44].

#### Heightened factors

Heightened factors were those which might be found in relation to a range of presentations but were likely to be particularly prevalent and/or challenging in relation to SH. The struggles reported by paramedics with accessing appropriate care pathways (including care from GPs and Crisis Teams) which might provide a safe and effective alternative to conveyance to hospital, can also be found in relation to other patient presentations, such as falls [45] and severe hypoglycaemia [46].

The challenges associated with dealing with patients who had consumed alcohol are another an issue found more widely in paramedic work [41], but are particularly relevant to SH. Previous research has identified a strong association between SH, suicidal behaviour [47] and alcohol, with Haw et al (2005) [48] reporting that up to 46.1% of SH patients have consumed alcohol within six hours prior to SH. SH patients who are intoxicated may present particular challenges in their interaction with paramedics, and may also become ineligible for certain referral pathways.

Paramedics’ descriptions of their vulnerability to acts of aggression and violence following SH fit with previous research, which found that more than 75% of ambulance personnel have experienced threats and/or violence when performing their duties, and of these, 67% were subjected to violence and 17% threatened with a weapon [49]. In SH however, such fears of violence are heighted amongst paramedics, as it was reported in this study that patients are often intoxicated, confused, lack mental capacity and weapons used to inflict SH such as knives and broken bottles are present and may be used on the paramedic. An elevated risk of abuse and violence associated with SH has previously been reported elsewhere [50].

Compassion fatigue can occur when practitioners become emotionally exhausted and lose the ability to respond empathically to their patients [51]. While compassion fatigue might be found in any are of paramedic work, paramedics described it in relation to SH as being shaped not just be exhaustion but also by the fundamentally challenging nature of SH as a condition.

#### Factors specific to SH

The third set of factors influencing decision making are those which are specific to SH. Paramedics described feeling confused over the blurring of physical and mental health needs in SH, and uncertainty over whether a patient who deliberately harms themselves is suffering from a mental condition. People who SH and present to emergency carers also tend to experience multiple tensions, both psychological and social in nature [52]. Such complexity has been found in ambulance service presentations of SH [40], and makes it challenging for services to respond to people who SH [53]. There is a risk, however, of diagnostic overshadowing, where a focus on a person’s mental health diagnosis overrides the consideration of their physical health needs [54], and it can compromise patient safety [55].

Paramedics’ reported difficulty in understanding why people SH revealed how it challenged their role as care givers and preservers of life. SH is at odds with ‘sick role’ as set out by Parsons (1958) [56] which outlines a shared societal understanding that those who are sick are not responsible for their status, but do have a responsibility to try to get better. This disjunction may lead paramedics to make judgements about SH patients’ value [57], regardless of clinical need. Finally, the care delivered to patients who SH is overshadowed by anxieties about future risk of suicide, implicit in all decision making about care for this group.

Paramedics reported the need for education in caring for people who SH. Whilst five of the paramedics interviewed had received education specific to SH, all of the paramedics interviewed reported the need for further the training education in this area. This lack of training and education is consistent with previous studies reviewed in the metasynthesis and systematic review previously conducted by the authors [8,11], which report between 75% to 90% of staff had not received any training.

### Strengths and limitation

The study was conducted in one ambulance service setting with a small sample of paramedics. Other grades of ambulance staff may be involved in care work with people who SH, but paramedics’ training and professional registration gives them added responsibilities. Participants were drawn from a diverse demographic, and their experience ranged between one and thirty-eight years of frontline paramedic care. This range of age and experience is considered a strength in the study, as we were looking for variety of experience and whether our emerging theories held true across this variety–this is one of the basic principles of GTM. The small sample size and variability of cross sectional design, may however be considered limitations, as there was a potential for selection bias, non-response bias and volunteer bias which may have occurred at particular points in time and service configuration.

NR as primary researcher was a paramedic, and also had significant influence over the research agenda. These issues were noted as a potential bias, as the researcher held a privileged position [58], but were counteracted by providing a detailed description of the role of EGTM, and undertaking activities such as systematic reviews and metasynthesis of the literature, coding checks and discussions with other members of the team who contributed to interpretation of interviews.

## Conclusion

It has been previously acknowledged that a major role of EMS systems is a safety net for persons with mental health problems [59]. Patients who SH frequently present to paramedics who identify them as a particularly problematic patient group. Many of the challenges and risks in caring for people who SH are present across the paramedic’s case mix. However, there are heightened, or specific to SH factors which make paramedic care for people who SH such a *wicked problem*, such as the challenges in assessing physical versus mental health problems, the role of alcohol and intoxication, and the potential for aggression and violence. The built-in complexities of SH collide with paramedics’ lack of education, confidence and competence in caring for people who SH, along with lack of referral options and support. All this is being played out in the acute presentation of SH, where significant harm or death by suicide are potential outcomes. Inappropriate detentions, negative attitudes and limited appropriate referral options may be adding to the significant numbers of people who SH that avoid care and are hidden to services which may be able to address their needs.

Paramedic care for people who SH is therefore a *wicked* and complex care interaction. It is unlike any other care scenario they encounter, as SH contradicts the fundamental principles of their role as preservers of life. Paramedics struggle to deal with the way in which SH violates the ‘sick role’ and the responsibility of the patient to try to get well, as set out by Parsons (1958) [56]. The results of this study indicate an urgent need for improved training, referral options, and support for Paramedics caring for people who SH. There should also be consideration of changes to mental health legislation which acknowledges the role of Paramedics in the assessment and provision of care for people who SH.

## Supporting information

S1 File Interview guide. (DOCX)
