# Supplementary material for: Paramedics’ perceptions of the care they provide to people who self-harm: A qualitative study using evolved grounded theory methodology
Source: PLoS One. 2018 Oct 17;13(10):e0205813. doi: 10.1371/journal.pone.0205813 (PMC6192640; doi:10.1371/journal.pone.0205813)
Supplement: S1 File — (DOCX) [file pone.0205813.s001.docx]

|  | Interview Guide |
| --- | --- |
| Pre Amble | My name is XXX I want to thank you for taking the time to meet with me today. I would also like to thank you for reading and completing the forms sent to you. We are here to discuss paramedics’ perceptions of care for those who Self Harm. Before starting the interview I would like you to consider the following definition of Self Harm. Please remember, this is just one definition of Self Harm:  *“An intentional act of self-poisoning or self-injury irrespective of the type of motivation or degree of suicidal intent. Thus it includes suicide attempts as well as acts where little or no suicidal intent is involved (e.g. where people harm themselves to reduce internal tension, distract themselves from intolerable situations, as a form of interpersonal communication of distress or other difficult feelings, or to punish themselves.*” (RCPsych 2010) |
| Questions: | 1. I have given you this definition so that when we discuss Self Harm we have a shared understanding. Would you agree with this definition? 2. What do you think about the care that you as a paramedic provide for people who Self Harm? 3. What do you think about the care that paramedics as a professional group give to people who Self Harm? 4. Can you give an example of an incident where a paramedic provided what you would perceive as a high standard of care to a person who had Self Harmed? 5. Can you give an example of an incident where a paramedic provided what you would perceive as a low standard to care to a person who had Self Harmed? 6. Is the care that paramedics give to people who Self Harm appropriate? 7. The care that those who Self Harm get in an emergency may affect their future view of care, case management or future Self Harm incidents. Do you feel paramedic care should be influence by these factors? 8. Do you feel your care is sufficiently informed and supported to deliver care for people who Self Harm? |
| Closing Key  Components:  • Additional  comments  • Next steps  • Thank you | Wrap up notes to self:   1. Do you have anything else to add? 2. Is there anything I should have asked? 3. How did the interview feel for you? 4. You have agreed to follow up interviews is this still OK with you? 5. Thank you for your time. |
